# Complete mitochondrial genome of *Gymnocanthus intermedius* and *Gymnocanthus herzensteini* (Scorpaeniformes: Cottidae)

**DOI:** 10.1080/23802359.2019.1642158

**Published:** 2019-07-22

**Authors:** Chang-Ho Yi, Hyuck Joon Kwun, Young Sun Song, Yung Kun Kim, Won Kim, Il-Hun Kim

**Affiliations:** a Department of Ecology and Conservation, National Marine Biodiversity Institute of Korea, Seocheon, Republic of Korea;; b College of Natural Sciences, Seoul National University, Seoul, Republic of Korea;; c College of Fisheries Science, Bukyong National University, Busan, Republic of Korea;; d Research Center for Endangered Species, National Institute of Ecology, Seocheon, Republic of Korea

**Keywords:** *Gymnocanthus intermedius*, *Gymnocanthus herzensteini*, Cottidae, mitochondria genome, Scorpaeniformes

## Abstract

Here, we report the complete mitochondrial genomes of the Sculpins species *Gymnocanthus intermedius* and *Gymnocanthus herzensteini*. The mitogenomes were determined to be 16,639 bp for *G. intermedius* and 16,691 bp for *G. herzensteini*. The mitogenomes comprised 13 protein-coding genes, 2 rRNA genes, 22 tRNA genes, and a non-coding region. We then used the mitogenome data to construct a phylogenetic tree for these two species and an additional three species within the order Scorpaeniformes.

The genus *Gymnocanthus* belongs to the family Cottidae (Nakabo 2013), contains nine species distributed in the northern coasts of the Pacific and the Atlantic Ocean including the adjacent Arctic (Froese and Pauly 2003; Nelson et al. 2016; Fricke et al. 2019). Among them, *Gymnocanthus intermedius* and *Gymnocanthus herzensteini* occurs in cold waters around Korea, Japan, and Russia of the western North Pacific, and are used as fishery resources (Kim et al. 2005; Yang et al. 2013).

In this study, a *G. intermedius* (Voucher No. MABIK PI00039374) and *G. herzensteini* (MABIK PI00039372) specimen for complete mitogenome sequencing was collected for complete mitogenome sequencing from the commercial fish market (N 37°33″, E 129°7″) near the East Sea of Korea. The fish were kept in a deep freezer deposited at National Marine Biodiversity Institute of Korea. We dissected the right dorsolateral muscle of the specimens for DNA extraction and preserved it in 95% ethanol. Genomic DNA was extracted with the Qiagen DNeasy Blood and Tissue kit (Qiagen Korea Ltd, Seoul, South Korea) following the manufacturer’s instructions. The complete mitochondrial DNA was sequenced using the Hiseq2000 platform with the next-generation sequencing technique (Illumina, San Diego, CA, USA). Geneious 9.1.3 (Biomatters Ltd, Auckland, New Zealand), tRNA Scan-SE1.21 software (http://lowelab.ucsc.edu/tRNAScan-SE/), and MitoFish (Mitochondrial Genome Database of Fish, http://mitofish.aori.u-tokyo.ac.jp/) were executed to assemble and annotate the mitochondrial DNA sequences.

The mitochondrial genome (GenBank accession no. KX148473) was 16,639 bp, and the base composition was 30.0% A, 10.0% T, 33.3% G, and 26.7% C in *G. intermedius*. A C + T rich (60.0%) feature was noted in *G. intermedius*. The *G. herzensteini* mitochondrial genome (GenBank accession no. KX148474) was 16,691 bp and consisted of 25.7% A, 28.6% T, 8.6% G, and 37.1% C. An A + T rich (54.3%) feature was also observed. Both genomes contained 13 protein-coding genes, 2 rRNA genes (12S and 16S RNA), 22 tRNA genes, and one non-coding region of a displacement loop region. The gene arrangement and transcription direction were identical to each other.

On the basis of the 13 protein-coding gene sequences of *G. intermedius* and *G. herzensteini*, a phylogenetic tree of the order Scorpaeniformes was reconstructed by using MEGAX (Kumar et al. 2018) (Figure 1). Our phylogenetic result was consistent with a previous phylogenetic classification of the order Scorpaeniformes based on phenotypic and molecular characteristics (Smith and Busby 2014; Fricke et al. 2019; Song et al. 2019).

**Figure 1.**
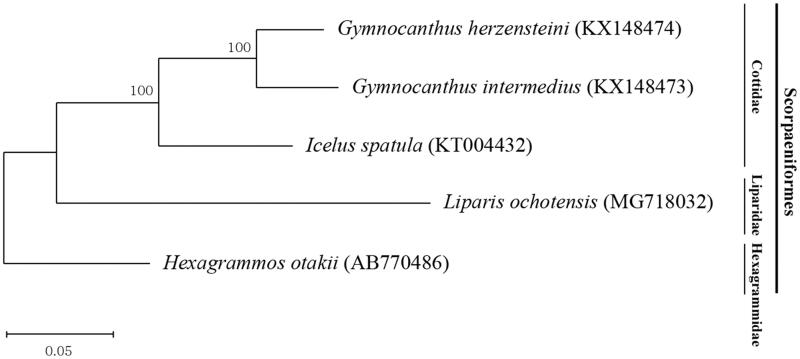
Maximum-likelihood (ML) tree reconstructed from five species belonging to the order Scorpaeniformes based on 13 protein-coding genes in the mitochondrial genome. The species belonging to family Hexagrammidae was usePaulyd as outgroup. The complete mitogenome sequences were downloaded from GenBank using the accession number indicated after the scientific name of each species. A bootstrap values above 50% in the ML analysis are indicated at each node.
